# Prenatal ultrasonography and Doppler sonography for the clinical investigation of isolated ventricular septal defects in a late second-trimester population

**DOI:** 10.1186/2047-783X-19-3

**Published:** 2014-01-24

**Authors:** Wei-Hsiu Chiu, Ming-Chon Hsiung, Ran-Chou Chen, Xiao-Min Xiao, Cai-Lin Wu, Tao-Hsin Tung

**Affiliations:** 1 Department of Biomedical Imaging and Radiological Sciences, School of Biomedical Science and Engineering, National Yang-Ming University, 112 Taipei, Taiwan; 2 1st Clinical Medical College, Jinan University, 510632 Guangzhou, China; 3 Department of Obstetrics and Gynecology, Chung Shan Hospital, 106 Taipei, Taiwan; 4 Division of Cardiology, Department of Medicine, Cheng-Hsin General Hospital, 112 Taipei, Taiwan; 5 Department of Radiology, Taipei City Hospital, 100 Taipei, Taiwan; 6 Department of Obstetrics and Gynecology, The 3rd Affiliated Hospital of Sun Yat-Sen University, Guangzhou, China; 7 Department of Medical Research and Education, Cheng Hsin General Hospital, No. 45, Cheng-Hsin Street, 112 Taipei, Taiwan; 8 Faculty of Public Health, School of Medicine, Fu-Jen Catholic University, 24205 New Taipei City, Taiwan

**Keywords:** Ultrasonography, Ventricular septal defects, Second trimester

## Abstract

**Background:**

The purpose of this study was to evaluate the efficacy of prenatal ultrasonography and Doppler sonography in detecting isolated ventricular septal defects (VSDs) in a late-second-trimester population.

**Methods:**

Fetal echocardiography, Doppler ultrasound, and biometry were used to evaluate 2,661 singleton fetuses (1,381 male fetuses and 1,280 female fetuses) between 1 August 2006 and 31 May 2010. The efficacy of each fetal biometry, Doppler ultrasound, and nasal bone length (NBL) measurement was evaluated in all of the fetuses. A standard fetal echocardiographic evaluation, including two-dimensional gray-scale imaging and color and Doppler color flow mapping, was performed on all fetuses.

**Results:**

We detected isolated VSDs in 124 of the 2,661 singleton fetuses between 19 and 24 weeks of gestation. The prevalence of isolated VSDs in the study population was 4.66%. A multiple logistic regression analysis indicated that short fetal NBL (odds ratio = 0.691, 95% confidence interval: 0.551 to 0.868) and the pulsatility index (PI) of the umbilical artery (odds ratio = 8.095, 95% confidence interval: 4.309 to 15.207) and of the middle cerebral artery (odds ratio = 0.254, 95% confidence interval: 0.120 to 0.538) are significantly associated with isolated VSDs.

**Conclusion:**

Late-second-trimester fetal NBL, umbilical artery PI, and middle cerebral artery PI are useful parameters for detecting isolated VSDs, and can be used to estimate the *a priori* risk of VSDs in women at high risk and at low risk of isolated VSDs.

## Background

Prenatal ultrasound screening is widely employed for detecting congenital malformations and structural heart defects. In previous studies, the prevalence of fetal congenital heart disease (CHD) ranged from 3/1,000 to 10/1,000 total live births. In the presence of CHD, ventricular septal defects (VSDs) are highly prevalent in newborns [1-3]. VSDs account for 30 to 35% of all CHD detected after birth. Previous studies have identified a VSD prevalence of 10 to 16% in fetal tests [1,4,5]. VSDs were also associated with other heart structural defects, such as right ventricular outflow tract and left ventricular outflow tract anomalies, as well as some aspects of Tetralogy of Fallot. Chromosomal anomalies associated with a VSD include trisomies 21, 13, and 18, deletion in 22q11, and nonchromosomal malformed fetuses [6,7].

The incidence of VSDs is the highest and second highest in live births and stillbirths, respectively [1]. VSDs are also the most common CHD diagnosis within the first year of life and the most frequently detected prenatal heart defect. VSDs represent one of the major sources of false-negative diagnoses in the uterus. Improvements in prenatal screening and VSD diagnosis can reduce neonatal mortality and morbidity [8,9]. B-mode fetal echocardiography as well as M-mode and color-mode echocardiography are the clinical screening methods used for VSDs [10-12]. Previous studies have proposed fetal Doppler echocardiography screening as an additional screening tool for VSDs [13-15]. Other studies indicated a high correlation between results obtained using fetal echocardiography and color Doppler echocardiography, reporting high sensitivity and specificity, and a low false-positive rate for CHD in fetal echocardiography examinations [16-19]. Conducting Doppler assessments of fetal arterial blood flow might increase the rate of identification of fetuses at risk of CHD. According to previous studies, CHD is associated with a significantly decreased pulsatility index (PI) in the middle cerebral artery (MCA) and an increased PI in the umbilical artery (UA) [20-22].

Fetal VSD assessment is a major component of a prenatal fetal heart examination, and is a useful primary diagnostic and screening tool for fetal CHD or chromosomal anomalies. In this study, we investigated fetal variations in VSDs using fetal arterial Doppler assessments to establish the risk of fetal VSDs in the general population.

## Methods

### Study participants

The study participants were 2,661 women with singleton pregnancies who underwent a fetal level II prenatal ultrasound examination, including detailed fetal echocardiography, in one teaching hospital and three obstetric clinics in Taipei, Taiwan between August 2006 and May 2010. As part of their prenatal care routine, all women underwent a prenatal ultrasound (including echocardiography) at 32 to 34 weeks of gestation. All procedures were performed in accordance with the guidelines of our institutional ethics committee and the tenets of the Declaration of Helsinki. All patient information was anonymous. Access to personal records was approved by the Hospital Human Subjects Review Board at Fu-Jen University, Taipei, Taiwan.

The gestational age (GA) of a fetus was determined according to the date of confinement estimated by the patient. If the estimated date of confinement was unknown, the difference between the ultrasonically estimated GA and the determined GA was assumed to be <10 days. For all fetuses with any associated abnormality, an antenatal diagnosis was arranged at a medical center within 1 to 2 weeks. The study exclusion criteria included a previous history of chromosome abnormalities, fetal structural anomalies or other CHD, and maternal complications.

### Ultrasound equipment and intraobserver variability

All fetal examinations were performed using a Voluson730ProV ultrasound platform (General Electric Medical Systems, Milwaukee, WI, USA) with multifrequency transabdominal transducers. A single experienced registered sonographer collected all scans and fetal measurements. Reliability statistics were used to assess the agreement of intraobserver reliability among repeated fetal biometry measurements collected by the same examiner.

### Measurement of fetal nasal bone and biometric examination

Fetal nasal bone length (NBL) was measured in a midsagittal view of the head using appropriate image magnification. To prevent measurement error, if the nasal bone was absent or shortened then the angle of the fetal nose was maintained at between 45° and 135°. Each increment was set at a 0.1 mm caliper distance, at the appropriate image magnification, to maintain accuracy [23].

The biparietal diameter (BPD) was measured on a transverse axial section of the fetal head, which included the midline falx and the thalami, symmetrically positioned on either side of the falx from the outer edge of the nearer parietal bone to the inner edge of the more distant parietal bone, and the septum pellucidum was visualized at one-third of the frontal–occipital distance [24].

The plane of the abdominal circumference (AC) was measured on a transverse section through the fetal abdomen. This plane was at the level of the liver and stomach bubble including the ductus venosus within the point of bifurcation of the main portal vein into its right and left branches in the anterior third of the fetal abdomen in the axial plane 90° to the fetal spine and posterior to the aorta. Measured from skin to skin, the transverse and anterior–posterior diameters were first measured as the long or short axis of an onscreen ellipse, and the circumference was obtained using elliptical calipers superimposed on the four points [24]. The single measurement plane of six limb bones (humerus, ulna, radius, femur, tibia, and fibula) was on the longest section of each structure. An appropriate magnification was used for the image to occupy three-quarters of the screen. The transducer was aligned to the long axis of the limb bone to obtain the appropriate plane of the section. The ossified portion of diaphysis was measured. The cartilaginous ends of the limb bones were excluded [25].

The estimated fetal body weight (FBW) was calculated using Hadlock’s formula [26]. This formula involves a summation of the BPD, AC, and femur length and provides a number in millimeters. The regression equation of the estimated FBW is:

$$\begin{array}{ll} {\mathrm{Log}}_{10}\left(\mathrm{FBW}\right)= & 1.4787-0.003343\left(\mathrm{AC}\times \mathrm{FL}\right)\\ & +0.001837{\left(\mathrm{BPD}\right)}^{2}+\left(0.0458\times \mathrm{AC}\right)\\ & +\left(0.158\times \mathrm{femur}\;\mathrm{length}\right) \end{array}$$

### Fetal heart assessment and Doppler measurement

The sonographers performed detailed fetal echocardiographic examinations of the 19-week to 24-week level II scans, under the direct supervision of an obstetrician. The detailed fetal heart assessment included basic screening and an extended cardiac scan [27,28]. The standard fetal echocardiographic evaluation involves employing all modalities of diagnostic ultrasound, including two-dimensional gray-scale imaging, and color and Doppler color flow mapping. The examination included the standard four-chamber view, the views of the left ventricular outflow tract and right ventricular outflow tract, the three-vessel view, and the basal short-axis two-dimensional B-mode echocardiography image.

**Figure 1:**
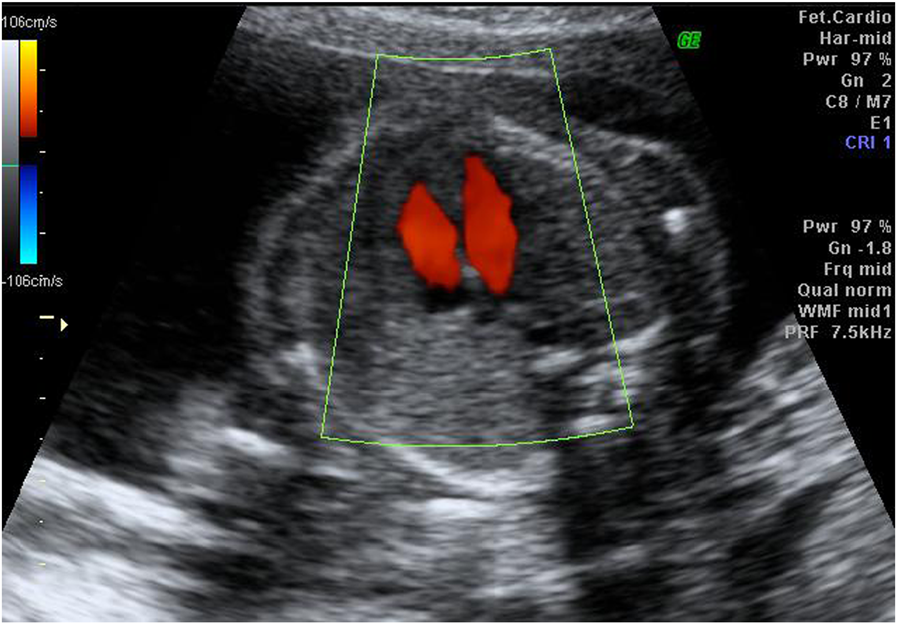
Color Doppler flow mapping.

**Figure 2:**
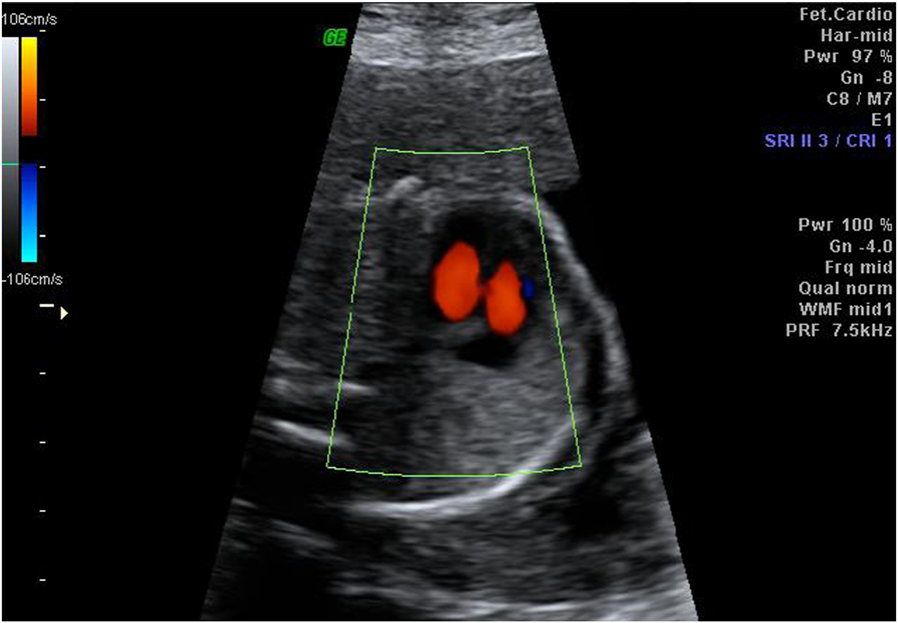
Shunt between the right and left ventricles.

Gray-scale imaging was initially performed, followed by color Doppler flow mapping (Figure 1). The high-pass filter was set at 100 Hz. The four-chamber view was observed using real-time scanning, and color Doppler was used to detect the presence of a VSD during the phase of a shunt between the right and left ventricles (Figure 2). The fetuses with VSDs that shunted flow were detected using two-dimensional color Doppler ultrasound examinations. The VSDs were recorded to ensure that the septum was perpendicular to the ultrasound beam, enabling the flow crossing the septum, indicating a septal defect, to be detected accurately [29]. For all fetuses with VSDs, a diagnostic antenatal echocardiographic examination was arranged at a medical center within 1 to 2 weeks. After delivery, the newborn babies with VSDs received a postnatal echocardiographic examination. In the non-VSD group, a prenatal ultrasound (including echocardiography) was performed at 32 to 34 weeks of gestation. A pediatrician examined the heart sound of all newborn babies.

**Figure 3:**
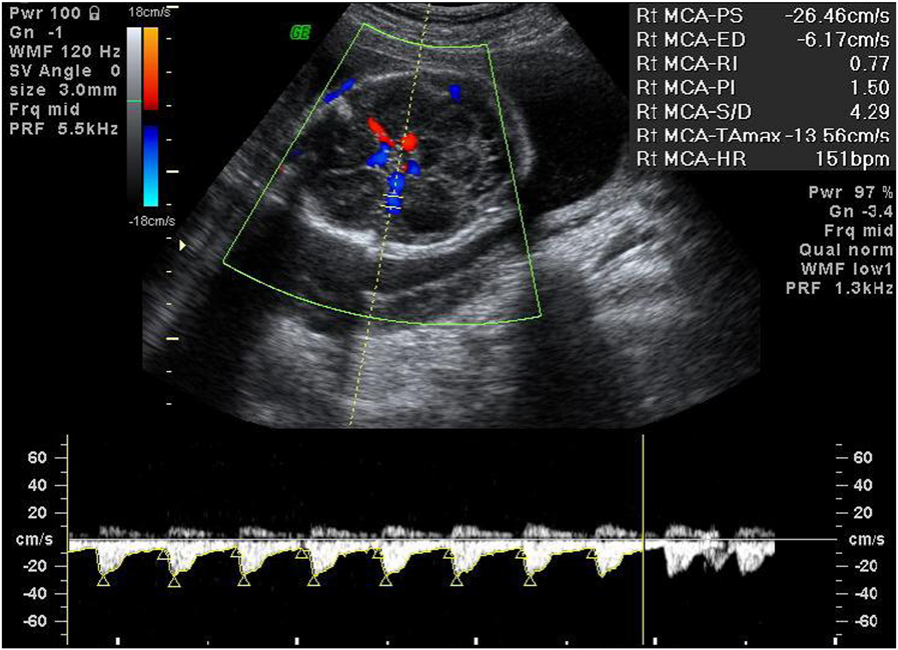
Middle cerebral artery.

**Figure 4:**
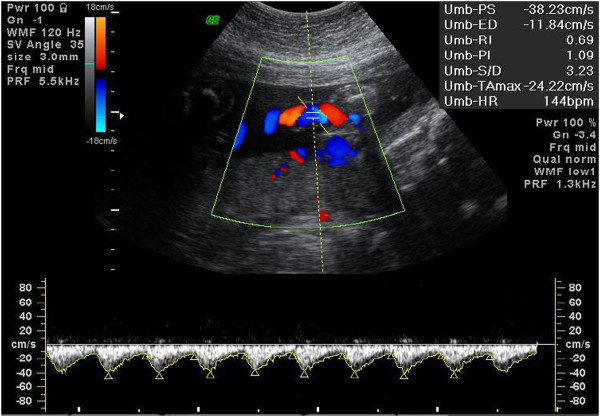
Umbilical artery.

An ultrasound examination for fetal biometry and a fetal heart assessment were conducted to exclude structural abnormalities. Liquor volume assessments, Doppler blood flow measurements of the UA, and Doppler blood flow velocity measurements of the MCA were also performed. All fetuses were subjected to Doppler examination of the MCA. An axial section of the brain, imaged in the BPD biometry plane, was obtained. The plane was then shifted downward toward the skull base. The circle of Willis was visualized and the MCA on one side was examined. The optimal site for recording a Doppler spectrum is the midpoint of the MCA, approximately 1 cm from the circle of Willis. The width of the sample volume was set between 2 and 4 mm. The wall filter was set as ≤120 Hz. Pulsed wave Doppler analysis was performed with the angle between the ultrasound beam and the direction of blood flow maintained as close to 0 to 25° as possible (Figure 3) [30,31]. The Doppler spectrum was sampled from a free loop of the umbilical cord with a wall filter setting >120 Hz (Figure 4) [30]. The PIs and resistance indices (RIs) of the UA and MCA Doppler waveform were measured.

In this study, the sensitivity of UA PIs >90% (1.47), MCA PIs <10% (1.23) and nasal bone length were 36.80%, 20.80% and 19.35%, respectively. The specificity of UA PIs >90% (1.47), MCA PIs <10% (1.23), and nasal bone length were 96.71%, 91.52%, and 93.46%, respectively.

### Data analysis

A statistical analysis was performed using the Statistical Package for the Social Sciences (Version 13.0 for Windows; SPSS, Chicago, IL, USA). *P* <0*.*05 was considered statistically significant. The mean, standard deviation, and percentiles of the GA, NBL, limb bone length, estimated FBW, cardiothoracic area ratio, cardiothoracic circumference ratio, PIs and RIs of the UA, and PIs and RIs of the MCA were determined. In addition, a logistic regression of a binary response variable (*Y*) on a continuous, normally distributed variable (*X*) with a sample size of 2,661 observations achieves 99% power at a 0.05 significance level to detect a change in Prob(*Y* = 1) from the value of 0.046 at the mean of *X* to 0.067 when *X* is increased to one standard deviation above the mean [32].

## Results and discussion

In this study, we evaluated 60 fetuses randomly selected during ultrasound screening. Table 1 presents the mean differences between the measurements collected using prenatal ultrasonography and Doppler sonography, and the accepted Cronbach’s alpha values. Except VSDs, we excluded 43 cardiac anomaly cases. The prevalence of all cardiac anomalies was 6.17% in our cohort. Because most of the parameters in studies change with gestational age, Table 2 presents the distribution of parameters as multiples of medians.

**Table 1:** Intraobserver repeatability of fetal biometry and Doppler assessment

	***n***	**Repeat measurements**	**Mean difference**	**SE**	**Cronbach’s α value**
Nasal bone length	60	2	−0.0033	0.06369	0.999
Biparietal diameter	44	2	−0.0591	0.13224	0.997
Head circumference	44	2	0.1682	0.12055	0.999
Humerus length	44	2	0.1045	0.06950	0.999
Radius length	44	2	0.0523	0.07045	0.998
Ulna length	44	2	0.0182	0.08455	0.998
Femur length	44	2	0.1182	0.07557	0.999
Tibia length	44	2	0.0318	0.06950	0.999
Fibula length	44	2	0.0295	0.08636	0.998
UA PI	60	2	0.0017	0.01659	0.999
UA RI	60	2	0.0010	0.00986	0.997
MCA PI	60	2	−0.0030	0.01816	0.997
MCA RI	60	2	0.0003	0.00367	0.999

MCA, middle cerebral artery; PI, pulsatility index; RI, resistance index; SE, standard error (difference in the paired measurements); UA, umbilical artery.

**Table 2:** Distribution of study parameters (multiples of medians)

	**19 weeks**	**20 weeks**	**21 weeks**	**22 weeks**	**23 weeks**	**24 weeks**
Nasal bone length	0.96	0.93	0.95	0.96	0.92	0.90
Biparietal diameter	0.94	0.99	0.98	0.99	0.99	0.97
Head circumference	0.97	0.98	0.98	0.99	0.99	0.98
Abdominal circumference	1.00	0.99	0.98	0.96	0.99	0.99
Femur length	0.99	0.94	0.97	0.97	1.00	0.98
Tibia length	0.98	0.97	0.97	0.98	0.99	0.97
Fibula length	1.00	0.97	0.97	0.98	0.99	0.97
Humerus length	0.95	0.99	0.99	0.98	0.97	0.98
Radius length	0.99	0.96	0.98	0.98	0.98	0.95
Ulna length	1.00	0.99	0.99	0.99	0.99	0.97
Estimated FBW	0.97	0.96	0.96	0.94	0.97	0.99
UA PI	1.60	1.06	1.04	1.06	1.06	1.06
UA RI	1.22	1.05	1.03	1.03	1.01	1.03
MCA PI	0.88	0.95	0.98	0.90	0.92	0.89
MCA RI	0.94	1.00	0.99	0.96	0.98	0.96
MCA/UA PI ratio	0.61	0.93	0.99	0.88	0.99	0.99
MCA/UA RI ratio	0.77	0.98	1.00	0.95	0.96	0.98

FBW, fetal body weight; MCA, middle cerebral artery; MCA/UA PI ratio, ratio of middle cerebral artery resistance index to umbilical artery pulsatility index; MCA/UA RI ratio, ratio of middle cerebral artery resistance index to umbilical artery resistance index; PI, pulsatility index; RI, resistance index; UA, umbilical artery.

As shown in Table 3, the overall prevalence of VSDs in the screened population was 4.66%. A chi-square test indicated that the prevalence of VSDs exhibited a borderline statistically significant decrease as the GA increased (*P =* 0.06). The prevalence of VSDs in the male and female fetuses showed nonsignificant differences (4.85% vs. 4.45%, *χ*^2^ = 0.15, *P* = 0.70). After stratifying the data on GA into six age groups, the male fetuses exhibited a higher prevalence of VSDs than the female fetuses did in all GA groups except the 19-week to 23-week subgroup. The chi-square test indicated that the GA-specified prevalence of VSDs was significantly associated with age in the male (*P* = 0.04), but not the female (*P* = 0.38), fetuses.

**Table 3:** **Gender and gestational age specific prevalence of ventricular septal defects among screened subjects (*n*** **= 2,661)**

**GA (weeks)**	**Male**				**Female**				**Total**				
		**Screened ( *n *)**	**VSD ( *n *)**	**Prevalence (%)**	***P *** **value for trend test**	**Screened ( *n *)**	**VSD ( *n *)**	**Prevalence (%)**	***P *** **value for trend test**	**Screened ( *n *)**	**VSD ( *n *)**	**Prevalence (%)**	***P *** **value for trend test**
19^+0^ to 19^+6^	34	0	0.00		48	4	8.33		82	4	4.87		
20^+0^ to 20^+6^	182	11	6.04	0.04	170	7	4.12	0.38	352	18	5.11	0.06	
21^+0^ to 21^+6^	338	24	7.10		284	11	3.87		622	35	5.63		
22^+0^ to 22^+6^	382	20	5.23		340	17	5.00		722	37	5.12		
23^+0^ to 23^+6^	263	7	2.66		270	15	5.56		533	22	4.13		
24^+0^ to 24^+6^	182	5	2.74		168	3	1.79		350	8	2.29		
Total	1,381	67	4.85		1,280	57	4.45		2,661	124	4.66		

GA, gestational age; VSD, ventricular septal defect.

Table 4 presents the characteristics of the fetuses with and without VSDs in the late-second-trimester population. Using the two-sample independent *t* test, we identified that the GA, NBL, BPD, AC, femur length, tibia length, fibula length, humerus length, ulna length, radius length, estimated FBW, UA PI, UA RI, MCA PI, MCA RI, ratio of the MCA RI to UA PI, and ratio of MCA RI to UA RI were all significantly associated with VSDs.

**Table 4:** **Characteristics of ventricular septal defect (VSD) among screened subjects (*n*** **= 2,661)**

**Variable**		**Normal (*n*** **= 2,537)**		**VSD (*n*** **= 124)**	***P *** **value**
	**Mean ± SD**	**10 to 90%**	**Mean ± SD**	**10 to 90%**	
NBL (mm)	6.74 ± 0.87	5.60 to 7.90	6.40 ± 0.79	5.30 to 7.40	<0.0001
BPD (mm)	54.33 ± 9.10	48.00 to 60.00	52.65 ± 4.87	47.12 to 59.70	0.041
HC (mm)	197.34 ± 15.57	177.00 to 218.00	193.94 ± 14.66	175.00 to 213.40	0.017
AC (mm)	177.08 ± 16.15	156.00 to 198.00	171.36 ± 16.12	150.82 to 194.45	<0.0001
Femur length (mm)	37.33 ± 3.86	32.50 to 42.40	35.93 ± 3.78	30.72 to 41.10	<0.0001
Tibia length (mm)	32.92 ± 3.42	28.30 to 37.30	31.90 ± 3.53	27.12 to 36.92	0.001
Fibula length (mm)	32.76 ± 3.33	28.35 to 37.00	31.71 ± 3.27	26.88 to 36.00	0.001
Humerus length (mm)	35.39 ± 3.34	31.00 to 39.70	34.40 ± 3.24	29.94 to 38.70	0.001
Radius length (mm)	30.34 ± 2.94	26.70 to 34.00	29.38 ± 2.70	25.70 to 32.46	<0.0001
Ulna length (mm)	32.92 ± 3.21	28.80 to 37.00	32.07 ± 3.14	28.00 to 36.30	0.004
Estimated FBW (g)	508.75 ± 116.48	364.00 to 666.00	470.74 ± 107.31	343.60 to 608.20	<0.0001
CTAR	0.22 ± 0.12	0.18 to 0.25	0.21 ± 0.03	0.18 to 0.25	0.753
CTCR	0.46 ± 0.06	0.42 to 0.50	0.46 ± 0.03	0.43 to 0.50	0.131
UA PI	1.16 ± 0.22	0.93 to 1.40	1.33 ± 0.34	0.99 to 1.91	<0.0001
UA RI	0.69 ± 0.08	0.60 to 0.78	0.74 ± 0.09	0.64 to 0.86	<0.0001
MCA PI	1.59 ± 0.27	1.26 to 1.90	1.48 ± 0.24	1.18 to 1.82	<0.0001
MCA RI	0.81 ± 0.08	0.72 to 0.91	0.79 ± 0.65	0.71 to 0.87	0.008
MCA/UA PI ratio	1.41 ± 0.34	1.02 to 1.79	1.19 ± 0.38	0.66 to 1.67	<0.0001
MCA/UA RI ratio	1.19 ± 0.18	0.99 to 1.40	1.09 ± 0.18	0.85 to 1.30	<0.0001

AC, abdominal circumference; BPD, biparietal diameter; CTAR, cardiothoracic area ratio; CTCR, cardiothoracic circumference ratio; FBW, fetal body weight; HC, head circumference; MCA, middle cerebral artery; MCA/UA PI ratio, ratio of middle cerebral artery resistance index to umbilical artery pulsatility index; MCA/UA RI ratio, ratio of middle cerebral artery resistance index to umbilical artery resistance index; NBL, nasal bone length; PI, pulsatility index; RI, resistance index; SD, standard deviation; UA, umbilical artery; VSD, ventricular septal defect.

We then used a multiple logistic regression model to evaluate the effects of independent associated risk factors on VSDs. Table 5 presents the NBLs (odds ratio = 0.69, 95% confidence interval: 0.55 to 0.87), UA PIs (odds ratio = 8.10, 95% confidence interval: 4.31 to 15.21), and MCA PIs (odds ratio = 0.25, 95% confidence interval: 0.12 to 0.54) following adjustment for confounding factors.

**Table 5:** **Multiple logistic regression of associated factors for ventricular septal defects among screened fetuses (*n*** **= 2,661)**

**Variable**	**β**	**SE**	**Ventricular septal defects (yes vs. no)**		***P *** **value**
			**Odds ratio**	**95% confidence interval**	
NBL	−0.37	0.12	0.69	0.55 to 0.87	0.001
UA PI	2.09	0.32	8.10	4.31 to 15.21	<0.0001
MCA PI	−1.37	0.38	0.25	0.12 to 0.54	<0.0001

MCA, middle cerebral artery; NBL, nasal bone length; PI, pulsatility index; UA, umbilical artery; SE, standard error.

Other independent variables included the GA, NBL, BPD, AC, femur length, tibia length, fibula length, humerus length, ulna length, radius length, estimated FBW, UA PIs, UA RIs, MCA PIs, MCA RIs, ratio of the MCA RI to UA PI, and ratio of MCA RI to UA RI, excepting the cardiothoracic area ratio and cardiothoracic circumference ratio.

According to our research, few studies have evaluated the parameters of circulation in fetuses with VSDs in Taiwan. Therefore, the purposes of our study were to evaluate the effects of isolated VSDs on fetal arterial hemodynamics and to determine the prognostic value of fetal arterial Doppler sonography in fetuses with VSDs. The prevalence of intrauterine growth restriction in the VSD and non-VSD groups was estimated as 4.84% and 4.46%, respectively. These results implied that fetuses with isolated VSDs were significantly smaller than the fetuses with normal cardiac anatomy. In addition, our study results indicated that the hemodynamics of the UA and MCA in all fetuses with CHD differed significantly from those of healthy fetuses. These findings support those of previous studies [20-22]. However, from a clinical viewpoint, abnormal UA PIs, UA RIs, MCA PIs, and MCA RIs are no more frequent in fetuses with isolated VSDs than in healthy fetuses. Our results indicated that the UA PI and the MCA PI represent risk factors for fetal VSDs. This finding is consistent with those of other population-based studies [20,22,33].

In this study, the fetuses with VSDs exhibited significantly lower values for fetal biometry and FBW than did the fetuses without VSDs. In Mari and Deter’s study, reductions in the fetal MCA PI were associated with perinatal growth restriction [34]. Lower fetal MCA PI values indicate that the human fetus responds to hypoxemia by centralizing blood flow to the brain in a process known as the brain-sparing effect [35]. A sequence of hemodynamic changes occurs as the fetal condition progressively deteriorates; however, the precise nature of these changes has not been fully elucidated.

Our study results indicated that the risk of fetal VSDs decreases with the degree of NBL shortening. VSDs are the most common lesion in trisomy 21; therefore, identifying fetal VSDs might increase the identification rate of fetuses at risk of developing Down syndrome [36]. Down syndrome is strongly associated with the hypoplastic fetal NBL, and fetal NBL examination is associated with high sensitivity, high specificity, and a low false-positive rate in Down syndrome screening [37,38]. In this study, we followed up the infants with VSDs for 24 months. Most of the infants’ VSDs were spontaneous closures between 6 and 18 months, and 28.6% (36/124) of the cases were spontaneous closures after delivery. In previous studies, the incidence of live-born infants with VSDs ranged from approximately 1 to 4.36% [39,40]. In addition, the proportion of VSD cases in the study population was 4.66%, which is higher than the previously reported rates. A possible reason for this discrepancy is natural fetal loss or pregnancy termination because of abnormalities or malformations of the fetus. Research showed that 74% of isolated fetal VSDs close spontaneously prior to birth [41].

One of the major limitations of this study was the potential for selection bias caused by the use of a screened hospital-based population. However, because of our relatively large sample size, we were able to achieve statistically significant conclusions. Secondly, we did not explicitly consider the sensitivity and specificity of the screening tests. Although the agreement of interobserver reliability seemed acceptable, nondifferential misclassification-bias identification still could have occurred. Thirdly, from the screening cost viewpoint, it is very difficult to follow all of the study cases with postnatal echocardiography. Finally, we collected our measurements at a single point in time, which could have eliminated the influence of various long-term demographic or biochemical factors on the risk of VSDs. Further longitudinal investigations to confirm our study findings are warranted.

## Conclusions

In this study we evaluated the NBL, MCA PI, and UA PI of fetuses with VSDs in the late-second trimester of pregnancy. Our results indicate that the evaluation of these parameters provides an appropriate method for the screening of fetal VSDs.

## Abbreviations

BPD: biparietal diameter; AC: abdominal circumference; CHD: congenital heart disease; FBW: fetal body weight; GA: gestational age; MCA: middle cerebral artery; NBL: nasal bone length; PI: pulsatility index; RI: resistance index; UA: umbilical artery; VSD: ventricular septal defect.

## Competing interests

The authors declare that they have no competing interests.

## Authors’ contributions

W-HC and T-HT executed the study and drafted the manuscript. M-CH, R-CC, and T-HT participated in the study design and performed the statistical analyses. M-CH, R-CC, X-MX, and C-LW envisioned the study and coordinated drafting the manuscript. All authors read and approved the final version of the manuscript.
